# For better or worse: crosstalk of parvovirus and host DNA damage response

**DOI:** 10.3389/fimmu.2024.1324531

**Published:** 2024-02-23

**Authors:** Songbiao Chen, Feifei Liu, Aofei Yang, Ke Shang

**Affiliations:** ^1^ Laboratory of Functional Microbiology and Animal Health, College of Animal Science and Technology, Henan University of Science and Technology, Luoyang, Henan, China; ^2^ Luoyang Key Laboratory of Live Carrier Biomaterial and Animal Disease Prevention and Control, Henan University of Science and Technology, Luoyang, Henan, China; ^3^ The Key Lab of Animal Disease and Public Health, Henan University of Science and Technology, Luoyang, China; ^4^ Ministry of Education Key Laboratory for Animal Pathogens and Biosafety, Zhengzhou, Henan, China

**Keywords:** parvovirus, host, DNA damage response, NS1, replication

## Abstract

Parvoviruses are a group of non-enveloped DNA viruses that have a broad spectrum of natural infections, making them important in public health. NS1 is the largest and most complex non-structural protein in the parvovirus genome, which is indispensable in the life cycle of parvovirus and is closely related to viral replication, induction of host cell apoptosis, cycle arrest, DNA damage response (DDR), and other processes. Parvovirus activates and utilizes the DDR pathway to promote viral replication through NS1, thereby increasing pathogenicity to the host cells. Here, we review the latest progress of parvovirus in regulating host cell DDR during the parvovirus lifecycle and discuss the potential of cellular consequences of regulating the DDR pathway, targeting to provide the theoretical basis for further elucidation of the pathogenesis of parvovirus and development of new antiviral drugs.

## 1 Introduction

Parvoviridae members are single-stranded (ss) DNA virus with a genome size of approximately 5.0 kb, containing two inverted terminal repeats (ITRs) on both sides of the genome (1, 2). The parvoviruses have no envelope and have a regular icosahedral capsid with a diameter of 18–26 nm. It can be widely spread in humans and many other species. The Parvoviridae is composed of three subfamilies: *Parvoviridae*, *Hamaparvovinae*, and *Densovirinae* (3, 4). The Parvoviridae subfamily comprises three genera: the *Parvovirus* genus, the *Erythrovirus* genus, and the *Dependovirus* genus.

The natural mode of infection spectrum of parvovirus is broad, including pigs, cattle, chickens, ducks, geese, dogs, cats, mice, and humans as well. Research studies in this area during the recent years has found that spillover of canine parvovirus type 2 (CPV-2) to pigs and pangolins (5, 6), different species of parvovirus have different receptors to infect the host, so it’s unclear how they can effectively implement a cross-species transmission method. The parvovirus genome encodes two open reading frames (ORFs), with the ORF1 on the left predominantly encodes the non-structural proteins NS1 and NS2, which are called Rep in adenovirus (AAV), and ORF2 on the right encodes the structural proteins VP1 and VP2. NS1 is the most crucial multifunctional protein of parvovirus, with binding specific site DNA activity, ATPase, endonuclease, and helicase activity (7–10). In addition, NS1 is closely related to host cell apoptosis (11), cycle arrest (12), and tissue damage (13) during viral infection and plays an indispensable role in the pathogenesis of parvovirus.

DNA damage response (DDR) is one of the fundamental physiological mechanisms in eukaryotes aimed at protecting the integrity of biological genomes (14). DDR is a highly conserved mechanism within the cells to resist DNA damage induced by external and internal factors. It is a complex regulatory network composed of multiple signaling pathways to monitor and transmit damage signals and can makeup appropriate response mechanisms. The DDR is required for the maintenance of genomic stability in response to endogenous and exogenous threats, which is a network of DNA surveillance pathways characterized by the activation of three central serine-threonine kinases that belong to the PIKK (phosphatidylinositol-3 kinase-related kinase) family: ATM (ataxia telangiectasia mutated), ATR (ataxia telangiectasia and Rad3-related), and DNA-PKcs (DNA-dependent protein kinase catalytic subunit) (15). It is vital in maintaining precise DNA replication and everyday life activities in the respective host cells.

Viruses utilize many strategies to manipulate the host pathways and hijack host types of machinery for efficient replication. Many DNA and RNA viruses are reported to interact with proteins involved in DDR, such as HIV-1 (16), pseudorabies (17), chikungunya virus (18), autographa californica multiple nucleopolyhedro (19), human papillomavirus (20) and porcine enteric coronavirus PEDV (21). Previous studies have shown that DNA damage signaling is required for parvovirus replication (22–27), and NS1 plays an essential role in this process. Therefore, this review aims to systematically appraise the interaction and mechanism between parvovirus NS1 and host cell DDR to provide a theoretical basis for further elucidating the cross-species transmission of parvovirus and the pathogenic mechanism and development of the new antiviral drugs.

## 2 Biological function of parvovirus NS1 protein

### 2.1 Replication cycle of parvovirus

**Figure 1:**
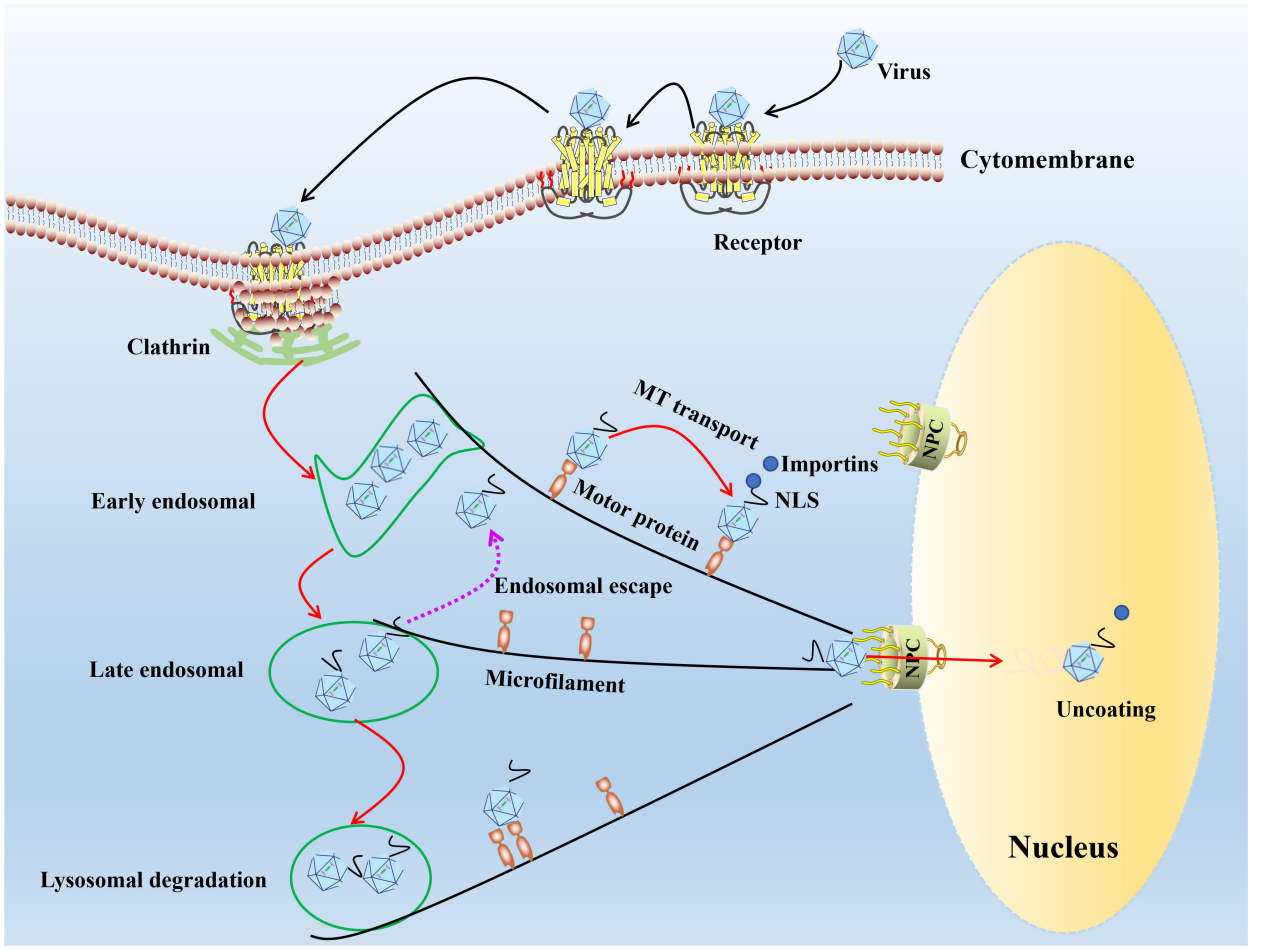
The model diagram of parvovirus entry cell and nuclear import. In the first step of infection, the viral capsid binds to receptors on the cell surface, and through endocytosis mediated by grid proteins, the conformation of the capsid protein changes under acidic conditions in the inner body. Most of the infected virus particles are transported to the degrading lysosome and accumulate for degradation in the lysosome. A small number of viral particles escape from the endosome and use the cytoskeleton and motion proteins to move near the nucleus, entering the nucleus through a nuclear pore dependent manner, initiating viral DNA replication within the nucleus.

The prerequisite for successful establishment of infection of the virus is bind to receptors on the cell surface (28, 29). Parvoviruses attach to host cells using a variety of cell surface receptors. Porcine parvovirus (PPV) binds to sialic acid on the cell surface glycoprotein. It enters the cell through clathrin-mediated endocytosis and macrophage action pathways (30), feline parvovirus (FPV)/CPV uses sialic acid and transferrin receptor (31). The human parvovirus B19 (B19V) attaches to the erythrocyte P antigen (32) and promotes its entry into the cell through low-pH mediated interactions with globulin (33), adeno-associated virus-2 (AAV-2) recognizes a variety of target cell receptors, including heparin sulfate proteoglycan (34), αVβ5 integrin (35), basic fibroblast growth factor receptor-1 (36) and KIAA0319L (37). After receptor binding, many parvoviruses enter cells through clathrin-mediated endocytosis (30). Low endosomal pH induces conformation changes in parvovirus capsid structure, resulting in the exposure of VP1 N-terminal unique region (VP1-u), the capsid VP1-u of PPV, B19V, minute virus of mice (MVM), and CPV contains a phospholipase A2 (PLA2) motif and a nuclear localization sequence (NLS), capsid interaction with motor protein depended on microtubule or microfilament network mediate viral DNA entry nucleus (**Figure 1**).

Parvoviruses replicate through a “rolling hairpin” mechanism, and replication in target cells typically occurs late in the S phase and early in the G2 phase of the cell cycle (38). After the virus enters the nucleus, the virus uncoating and releases the ssDNA genome, using the host cell DNA replication platform, the end of the parvovirus genome contains the palindromic sequence, which can be folded into a stable hairpin structure to allow the primers required for its own replication at 3′OH of the left ITR (39), and then the complementary strand of the viral genome is synthesized through the principle of base complementation, and the single-stranded genome of the virus is converted into replicating DNA (23), and finally the double-stranded DNA molecule is used as a template. Synthesizing viral progeny genomes and allowing RNA polymerase II transcription to initiate gene expression, parvovirus replication is strictly dependent on cellular DNA polymerase (Pols), parvovirus DNA replication seems to require only one Pols to extend built-in primers, parvovirus genomes are reminiscent of primer ssDNA templates, which are used to indicate Polδ and cofactors, proliferating cell nuclear antigen (PCNA), replication factor C (RFC) and replication protein A (RPA) are sufficient to form and function as DNA strand extension complexes, requiring RFC to load PCNA onto DNA, allowing for the recruitment of Polδ, while RPA binds and stabilizes the ssDNA region before nascent strands (38, 40, 41).

A long-standing unanswered topic is how and where the capsid uncoating for gene expression before entering the nucleus (42, 43). Earlier studies have shown that in the infectious parvovirus particles, the capsid surface exposed the 5’ end 20-30 nucleotides (44–47). However, the role of these 5 ‘ends or NLS in the nuclear release of viral DNA is unknown. Although the molecular mechanism of capsid decomposition is not yet clear, recent research into the dynamics of CPV in nuclear capsid shows that slow-moving karyoplasm CPV capsid with fast-moving capsid-derived ingredients, including the composition of the residue or disintegrate protein. This suggests that the capsid is disintegrated upon entry into the nucleoplasm (30, 48). At present, there are two possibilities to speculate, one is the capsid may be conducted before getting into the nucleus genome DNA hulling preparation because divalent cations in the cytoplasm of consumption will enhance B19V capsid uncoating (49); the other one is dependent on endosome acidification lead to Ca^2+^ release and NE disruption, such as CPV, H-1PV or AAV-2 (50, 51). The uncoated ssDNA viral genome in the nucleus is transformed into the replicating form dsDNA expressing the viral NS protein. Viral DNA replicates within the nucleus, expresses viral NS and capsid proteins, and then packages the genome into an empty capsid. Finally, the mature virus is expelled from the infected cell (52).

### 2.2 Biological function of parvovirus NS1 protein

NS1 is a multifunctional phosphorylated protein with various physiological enzyme activities such as DNA polymerase, helicase, endonuclease, and ATPase (1), which plays an essential role in viral replication, gene expression regulation, host cell apoptosis, and cell cycle arrest.

#### 2.2.1 NS1 protein is involved in gene expression regulation

Virus activation areas are present in the C- and N-terminals of the parvovirus NS1 protein. These sections are strongly linked to the control of host genes and viral gene expression, in addition to being important in the regulation of the virus’s life cycle. The two primary ways that gene expression is regulated are transcriptional and post transcriptional. They activate or inhibit transcription initiation, thus controlling the number of proteins synthesized during translation (53). At the transcriptional level, the regulation of gene expression largely depends on the promoter (54).

The parvovirus genome has two overlapping coding regions, the left promoter p4 and the right promoter p38, which control the transcription of non-structural and structural proteins, respectively (55). Previous studies have shown that the NS1 protein contains a trans-activation domain (TAD2; ^523^SSFFNLITP^531^), which can make its own genes and structural protein genes active and transcriptional activation. In terms of participating in gene expression regulation, NS1 protein can transactivate capsid protein-related p38 transcription factors (56). MVM genome p38 is a transcription-related regulator. NS1 is an effective transcriptional activator, stimulating the modified long terminal repeat promoter and the p38 promoter (from MVM) to promote gene expression (57). In addition, the NS1 of MVM can interact with p38, significantly enhancing the activity of the p38 promoter and promoting the production of viral capsid protein. NS1 can also regulate the transcription of its p4 promoter in viral gene transcription (52) and activate the p38 promoter by specifically binding DNA, thus promoting viral protein expression (52). B19V NS1 protein plays a vital role in gene regulation, which can bind to the p6 promoter of the virus with the help of transcription factors Sp1/Sp3 and regulates the expression of viral genes (58, 59). The interaction of the NS1 protein with the cellular transcription factors Sp1 and Sp3 is achieved by direct binding to DNA sequence elements; NS1 protein interaction with the Sp1/Sp3 transcription factor can reduce the activity of the p6 promoter (10). It has been observed that NS1 stimulates P4 promoter expression by binding to left-end inverted terminal repeats and promoting genomic amplification (56). In addition, NS1 transactivates several other host genes, such as the regulation of tumor necrosis factor-alpha and p21/WAF1 through the activation of AP-1/AP-2 and Sp1 (60, 61). PPV NS1 protein can increase the expression levels of IL-6 and TNF-α in a dose-dependent manner. Meanwhile, the NS1 protein can induce IκBα phosphorylation, leading to NF-κB phosphorylation and nuclear translocation (62). The trans-activated p38 promoter found in CPV is closely associated with the N and C-termini of the nonstructural protein NS1 (7). The mutation of lysine 405 to serine in the NS1 protein of H-1 parvovirus caused the loss of transactivation activity of the late promoter p38, and the virus lost the ability to replicate (63).

#### 2.2.2 NS1 and viral replication

NS1 is the largest non-structural protein of parvovirus and is also named after replication enzyme (Rep78/68) (64). NS1 contains several conserved functional sites in its gene sequence, such as the nuclease domain, N-terminal helicase structural site, and ATP binding site (52). These functional domains are essential for the efficient replication of viral DNA. When the initiation of parvovirus replication, the nucleus of restructuring, and formation of the unique nuclear foci termed “autonomous parvovirus-associated replication” (APAR) bodies (65–67), NS1 colocalizes with replicating viral DNA within APAR bodies and accumulates host replication involved proteins such as PCNA, RPA, and DNA polymerases α and δ (65–67). Meanwhile, parvovirus can use NS1 to bind the viral origin directly, facilitating viral DNA replication (68). MVM replication occurs in virally-induced APAR bodies, which also contain replicating genomes, the viral replicator protein NS1, RPA, and DNA polymerases α, δ, and cyclin A. DDR is activated by MVM infection and is typified by the phosphorylation of H2AX, Nbs1, RPA32, Chk2, and p53. These proteins are recruited to MVM replication centers and co-localized with NS1; as cellular DNA damage accumulates, the virus spreads to newly damaged sites to amplify infection (69). ATM inhibitors limit MVM replication and improve cell cycle arrest, suggesting that DNA damage pathways promote viral replication, partly by promoting cell cycle arrest (23). NS1 acted as an adaptor protein in this process, rapidly employed the viral genome to the DNA damage site of the host cell by interacting with the viral genome and proteins related to DDR and promoting efficient viral replication (70).

NS1 also has the activity of endonuclease (71). The NS1 of B19V interacts with the origin of viral DNA replication during the rolling loop replication. After the completion of the rolling loop replication of the virus, the NS1 protein forms a specific linear DNA single-strand incision at a particular site. It enters the next round of rolling loop replication (10). At the same time, the NS1 protein is a necessary targeted protein for viral DNA replication. The NS1 protein cannot simply diffuse through the nuclear pore complex (NPC) due to its considerable molecular weight, and NLS are required to interact with importin-beta (impβ) and other proteins from the complex outside the nuclear membrane and bind through the NPC in order to complete replication in the nucleus (4). Meanwhile, we also found that PPV NS1 contained two nuclear export signals (NESs) at positions 283-291 and 602-608 amino acids (aa) (NES1/NES2) (72). The nuclear export activities of NES1 and NES2 can be blocked by Leptomycin B (LMB), confirming that NS1 protein export from the nucleus depends on chromosome region maintenance 1 (CRM1) pathway. It has been shown that NS1 protein promotes viral replication through its functional NESs and NLS at positions 256-274 aa to mediate CRM1-dependent nuclear export pathway and α/β mediated nuclear import pathway between nucleus and cytoplasm (72).

#### 2.2.3 NS1 is involved in inducing host cell apoptosis

Parvovirus can change the morphology of host cells after infection, and this morphological change is related to cell apoptosis induced by parvovirus (73). NS1 is crucial for virus-induced cytotoxicity (74), which can cause many cell deaths to promote virus transmission, lead to tissue damage, and even cause some secondary diseases.

**Figure 2:**
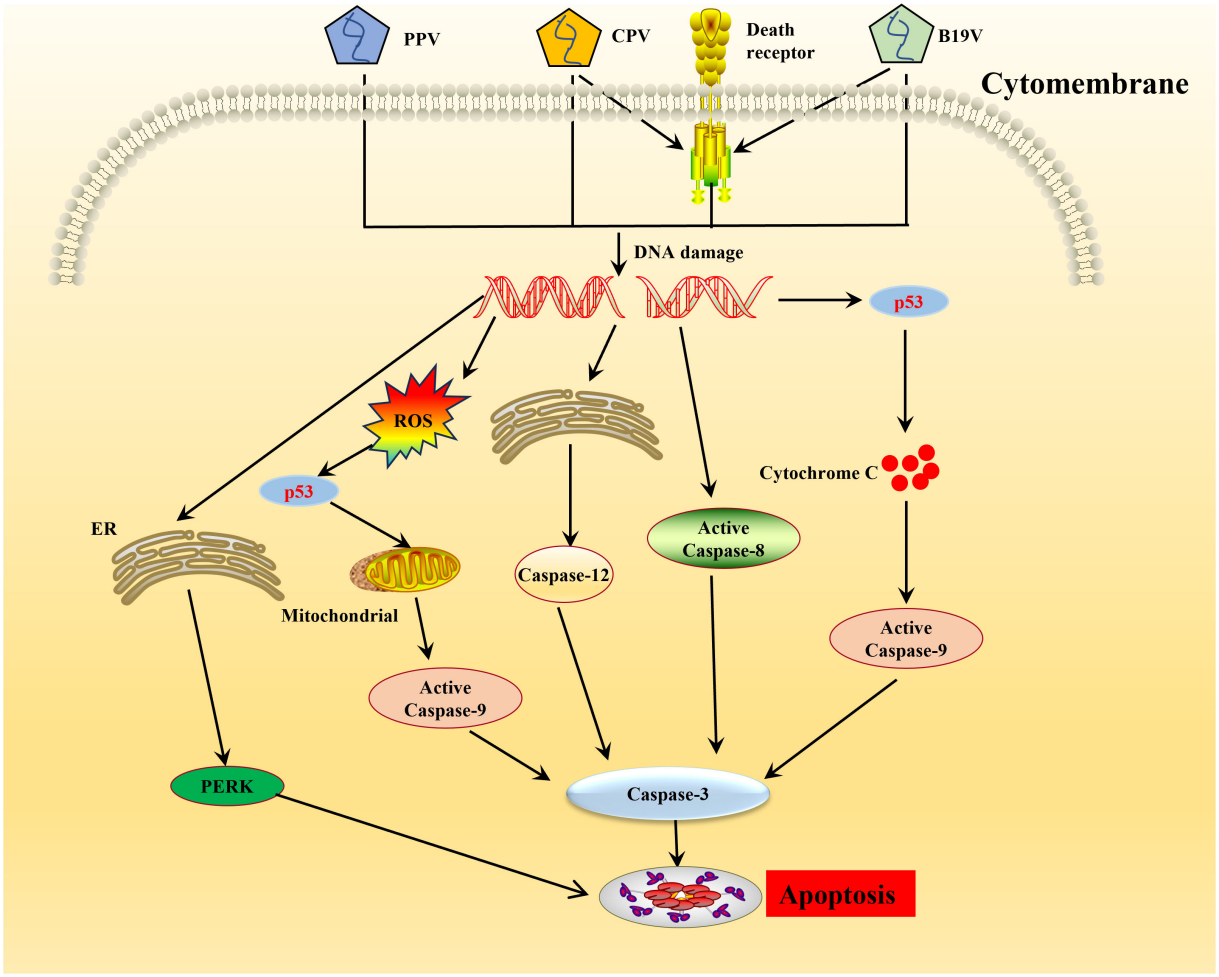
Schematic diagram of molecular mechanisms underlying the cell apoptosis induced by parvovirus. Once the parvovirus entry into the host cells, and causes DNA damage leading to activation of pro-apoptotic signals thereby creating oxidative stress, endoplasmic reticulum stress. This cellular stress causes a decrease in mitochondrial membrane permeability, the release of cytochrome C which further forms apoptosome complex with Apaf1, activating caspase 9, finally inducing cell death by intrinsic/mitochondrial pathway.

Recruitment of DDR kinases by viruses at the sites of DNA damage leads to cyclin-dependent kinases (CDKs) silencing and cell cycle arrest (75). Parvoviruses arrest cells in different stages depending on the host cell and virus type (69, 76–78). CPV causes DNA fragmentation and interferes with the cell cycle, producing time-increasing viral progeny, leading to the pathological consequences of infection and cell death following cell cycle arrest (79). ROS accumulation is considered the critical factor leading to DNA damage in the host cells. Cell cycle arrest caused by DNA damage and induction of cytochrome C release and proapoptotic molecules (Bax, p21, and p53) play an important role in the apoptosis induced by NS1 (13). PPV induces apoptosis in infected cells by activating caspase-9 and caspase-3, indicating that PPV-induced apoptosis occurs through an inherent apoptotic pathway (80–83). In PK-15 and PT cells, endoplasmic reticulum (ER) stress-induced apoptotic cell death inhibits PPV replication (84, 85) (**Figure 2**). Endoplasmic reticulum stress stimulates the unfolded protein response (UPR) pathway in cells, which is mediated by signaling pathways such as the protein kinase-like endoplasmic reticulum kinase (PERK) pathway and ultimately leads to cell apoptosis (84, 85) (**Figure 2**). The NS1 of B19V can significantly induce the phosphorylation levels of Cyclin A, Cyclin B1, and cell division Cyclin 2 (CDC2) during viral replication, resulting in up-regulation of CDC2-cyclin B1 complex kinase activity (86). This results in G2/M checkpoint stasis of the cell cycle, which induces cysteinyl aspartate specific proteinase (caspases) activation and DNA fragmentation, thus triggering apoptosis (87) (**Figure 2**). Apoptosis induced by CPV is attributed to ROS accumulation and activation of caspase-3, -8, -9, and -12, indicative that CPV-induced apoptosis occurs through extrinsic, intrinsic, and ER pathways (88, 89) (**Figure 2**). MEV induced apoptosis through the mitochondrial apoptosis pathway by activating p38 MAPK and the p53-mediated signaling pathway mediated by NS1 (90). In addition, some NS1 of parvoviridae members can interact with other host proteins to induce apoptosis in host cells. After infection with GPV, NS1 activates caspase-3 and caspase-9 by interacting with mitochondrial outer membrane porin VDAC, leading to apoptosis (91).

#### 2.2.4 NS1 participates in the host cell replication cycle

Parvoviruses cannot complete the replication cycle independently, and depend on host resources, and helper viruses are required for effective replication of AAV (92, 93). To make their replication cycle proceed smoothly, they need to continuously infect the host cell and use the replication mechanism of the host cell to replicate the viral genome to achieve the purpose of reproduction and survival. Many studies have shown that parvovirus NS1 can induce cell cycle arrest to promote the stable replication of the viral genome (**Table 1**). During MVM infection, the host cell cycle is blocked in the S phase and G2 phase through the mediation of p53 molecules (23). Meanwhile, NS1 was also found to be associated with cyclin-dependent kinase (CDKs) inhibitor p21CIP1, Cyclin A, CDK, Cyclin E, and CDK2 complexes, thereby mediating cell cycle arrest (78). Other studies have shown that the NS1 of B19 acts as a transcriptional trans-activator to activate ATR, thereby activating the ATR-CHK1-CDC25C-CDK1 pathway, blocking the cell cycle in the G2 phase and inducing cell apoptosis (94). The NS1 of CPV can induce cell cycle stagnation in the S phase, which is conducive to viral replication (78).

**Table 1:** Summary of cell cycle effects induced during parvovirus infection.

Virus	Viral proteins involved in the cell cycle	Cell cycle arrest phase	References
PPV	NS1	G1, G2	(13)
B19V	NS1,11kDa, VP1	S, G2, M	(24, 94–98)
HBoV	NP1	G2, M	(99)
CPV	NS1	G1, S	(100)
MVM	NS1	S, G2	(101)
H-1PV	NS1	G2, M	(102)

## 3 The regulatory role of host DDR response

DNA stores genetic information, and its damage and repair will directly affect the fate of the cells (103). Meanwhile, DNA also serves as a transcriptional template to guide protein biosynthesis and plays an indispensable role in the growth and development of organisms (104).

Cells are subjected to exogenous damage, such as different types of radiation or genetic agents, and endogenous damage, such as base depurination and deamination, during the transmission of cell genetic information. Cells respond to these adverse conditions by forming a complex network of signaling pathways known as DDR (105). This response is the basis for organisms to sense DNA damage signals, slow down or block cell cycle progression (cell cycle checkpoints), and activate different DNA repair or apoptosis mechanisms (106). DDR is a signal transduction pathway driven by protein phosphorylation, including direct repair (DR), base excision repair (BER), homologous recombination repair (HR), non-homologous end junction (NHEJ), nucleotide excision repair (NER), and mismatch repair (MMR) (107).

**Figure 3:**
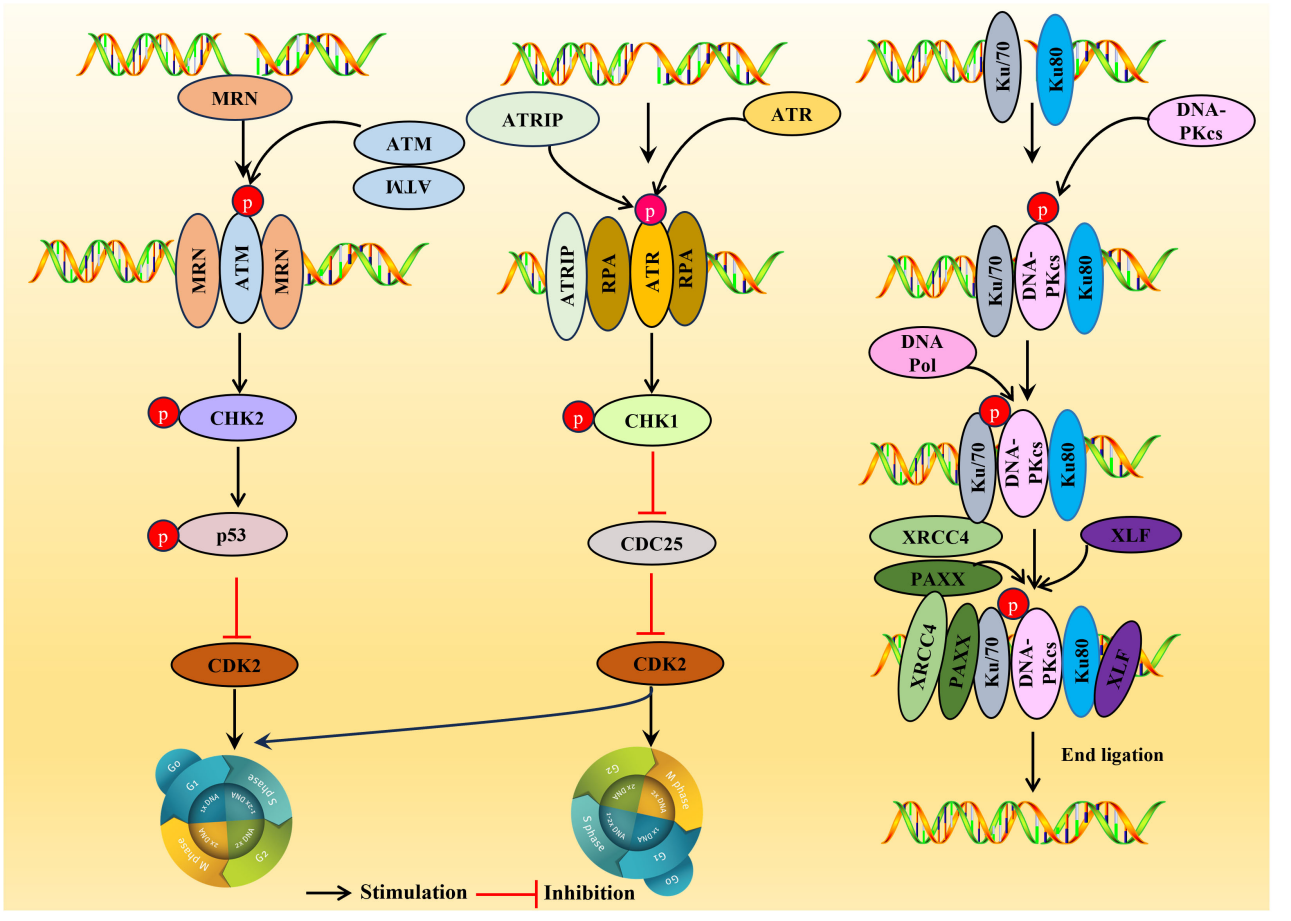
Overview of the DDR pathways. DDR, DNA damage response. ATM is usually located in the nucleus as a dimer (non-activated), and the activated ATM exists as a monomer. Upon sensing the DSB signal, the heterotrimer MRE11-RAD5-NBS1 (MRN) complex binds to the double-stranded DNA (dsDNA) terminal, prompting ATM and DSB sites to interact, resulting in ATM phosphorylation, then a large number of substates CHK2, p53 were activated and inhibiting CDK2 activity, the G1/S or G2/M cell cycle is arrested. ATR is activated in response mainly related to ssDNA. When there is DNA replication stress or DNA damage in the cell, the replication-associated protein A (111) wraps single-stranded DNA (ssDNA) and forms the RPA-ssDNA complex lead to ATR activation, ATR activation may lead to the activation CHK1, SMC-1, ATM and p21, which can regulate CDC25A, RAD51, p53 and DNA-PKCs, and is crucial for two important checkpoints in G1/S phase and G2/M phase. DNA-PK is activated usually in response to DSBs. In the DNA-PK complex, Ku70/Ku80 proteins endows the DSB terminal with high affinity and acts as an early sensor. Subsequently, DNA-PKcs was recruited into DSBs through Ku70/Ku80 proteins and triggering the activation of DNA-PKcs. After activation, DNA-PKcs recruit some host factors DNA Pol, XRCC4, PAXX and XLF to complete double stranded DNA repair.

DDR can induce the translocation of nuclear DNA into the cytoplasm, thereby triggering an immune response mediated by cytoplasmic DNA recognition receptors involved in innate immunity. DNA damage can induce considerable harmful effects, such as interfering with DNA replication, transcription, and even breaking, eventually leading to mutations and chromosome aberrations (108). DDR can protect the genomic integrity of cellular DNA during replication. When cells are threatened by endogenous damage and exogenous factors, they can eliminate the adverse consequences of DNA damage and prevent its transmission to daughter cells (109). DDR pathway is a DNA monitoring pathway network characterized by the activation of three major serine-threonine kinases belonging to the PIKK (phosphatidylinositol-3 kinase-related kinase) family: ATM (ataxia telangiectasia mutation), ATR (ataxia telangiectasia and Rad3 related), and DNA-PKcs (DNA dependent protein kinase catalytic subunit) (15, 110). ATM and DNA-PKcs are activated primarily in response to dsDNA breaks (DSBs), while ATR is activated in response to single-stranded DNA (ssDNA). There are, nonetheless, a lot of interconnections amongst these circuits. Recognition of DNA damage by the DDR kinases leads to the phosphorylation of numerous substrates that coordinate cell cycle arrest with the recruitment of DNA repair factors to sites of DNA damage (109) (**Figure 3**). Persistent DDR signaling can lead to apoptosis or senescence if the damage is too extensive or the lesions are not rapidly repaired.

### 3.1 ATM signaling pathways

ATM is a serine-threonine protein kinase and a critical regulatory factor in DDR. ATM is a true ‘commander’ involved in maintaining cell cycle checkpoints, repairing DNA damage, and maintaining telomeres in DNA double-strand breaks (DSBs), essential in regulating cell responses to DNA damage. ATM is usually activated in response to DSBs and is generally located in the nucleus as a dimer (non-activated), and the activated ATM exists as a monomer (**Figure 3**).

DSBs are the primary source of genomic instability, mainly processed through NHEJ or HR (109). Upon sensing the DSB signal, the heterotrimer MRE11-RAD5-NBS1 (MRN) complex binds to the double-stranded DNA (dsDNA) terminal, prompting ATM and DSB sites to interact, resulting in ATM phosphorylation. Consequently, a large number of substates CHK2 (15), p53 (112) were activated and inhibiting CDK2 activity, the G1/S or G2/M cell cycle is arrested (110), thus giving cells more time to repair DDR before entering mitosis (**Figure 3**).

### 3.2 ATR signaling pathways

ATR is activated mainly in response to ssDNA. When there is DNA replication stress or DNA damage in the cell, the replication-associated protein A (111) wraps single-stranded DNA (ssDNA) and forms the RPA-ssDNA complex, recruiting the ATR ligand ATRIP regulatory ATR activation; ATR activation may lead to the activation of a variety of downstream target molecules, such as the phosphorylation of CHK1, SMC-1, ATM, and p21 (113). Among them, CHK1 is the most important molecule, which can regulate CDC25A, RAD51, p53, and DNA-PKCs, and is crucial for two critical checkpoints in G1/S phase and G2/M phase (G1/S phase checkpoint governs the process of cells entering the DNA synthesis phase from a stationary state; G2/M checkpoint determines whether a cell splits in two to form two daughter cells) (114). Once ATR is activated, ATR phosphorylates CHK1, results in the phosphorylation and inactivation of CDC25 so that CDK2 cannot be activated, thereby blocking the G1/S or G2/M cell cycle (115).

### 3.3 DNA-PK signaling pathways

DNA-PK is a serine/threonine protein kinase complex, a 660 kDa holoenzyme complex composed of a DNA-binding catalytic subunit (DNA-PKcs) and a KU70/80 heterodimer. DNA-PK is expressed in almost all cells and is a key kinase in NHEJ repair (116). DNA-PK is usually activated in response to DSBs, and DNA-PK assembly at the end of DSB provides a platform for recruiting NHEJ factors. In the DNA-PK complex, Ku70/Ku80 proteins endow the DSB terminal with high affinity and act as an early sensor. Subsequently, DNA-PKcs were recruited into DSBs through Ku70/Ku80 proteins, triggering the activation of DNA-PKcs. After activation, DNA-PKcs recruit some host factors, DNA Pol, XRCC4, PAXX, and XLF, to complete double-stranded DNA repair (110).

## 4 Modulation of DDR pathway by parvovirus

DDR is an endogenous alert system that continuously detects genomic integrity to ensure accurate transmission of genetic information to daughter cells. The DDR mechanism protects the host genome from the harmful consequences of DNA breaks and identifies invading viral pathogens. DNA viruses replicating within the nucleus have evolved unique strategies to evade or usurp these DDR proteins. After infecting host cells, parvovirus can induce obvious DDR response (**Table 2**), but the specific mode and mechanism of action are still unclear. This section focuses on the role and mechanism of parvovirus NS1 in regulating host DDR response.

**Table 2:** Reaction between parvovirus and host DDR.

Virus	Protein	Approach	References
MVM	NS1	ATM	(117–120)
HBoV1	NS1	ATM, ATR, DNA-PK	(25, 121, 122)
AAV-2	Rep68/78	DNA-PK	(123, 124)
PPV	NS1	Unknown	(52)
MVC	Unknown	ATM, ATR	(125)
B19V	NS1	ATR, DNA- PK	(94, 126)

### 4.1 Role of NS1 in the regulation of host cell DDR

The integration of viral genome, DNA mismatch, or the influence of environmental physical and chemical factors can cause DNA damage and lead to genomic instability, which can induce diseases such as cancer. As a result, cells have evolved a complete DDR system to deal with these challenges. At the same time, because more viruses will cause host DDR after infecting host cells, viruses have developed relevant strategies to fight host DDR or use host DDR to complete its life cycle and carry out a “game” with the host (127–129).

**Figure 4:**
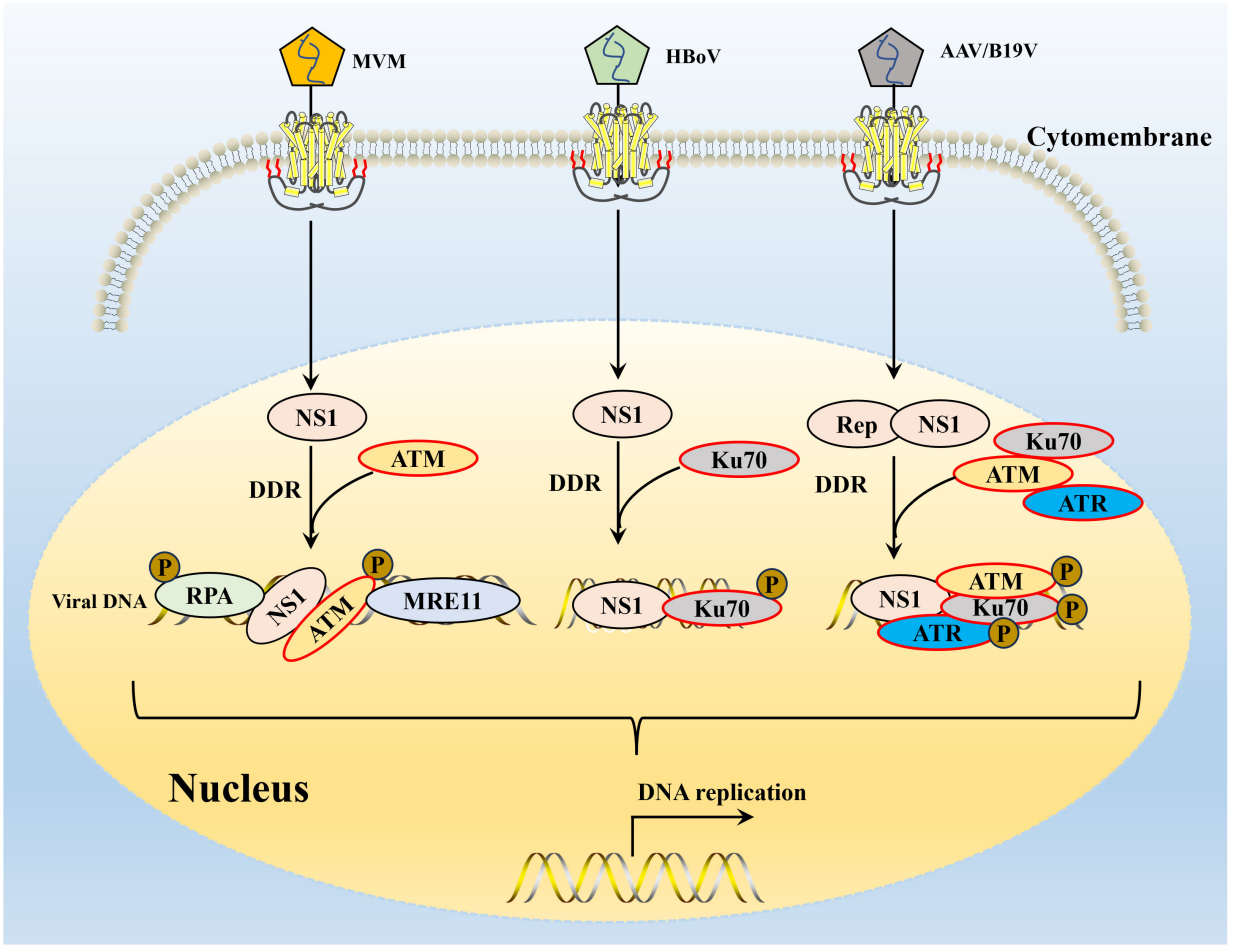
The interaction of parvovirus NS1 and host DNA damage response. DDR, DNA damage response. The NS1 of MVM induced DDR depends on signal transduction by ATM kinase and leads to ATR-mediated signal inactivation, targeted RPA 32 and MRE11 expression promote viral replication. After B19V or AAV infection, three major PI3K cascades are activated of NS1, resulting in the recruitment of Ku70, ATM and ATR to the viral DNA replication center and co-localization of DNA damage site. HBoV infection of human respiratory epithelial cells cultured at the gas-liquid interface can induce significant DDR, and Ku70 activation has been shown to be a key DDR pathway for HBoV DNA replication.

The ectopic expression of the viral NS1 protein also causes host cell replication stress and replication fork shortening because parvovirus replication exploits the many DNA replication forks that typically lie in a cell’s DNA damage signaling proteins (118). Further studies confirmed that NS1-induced replication stress depends on its DNA-binding function, as NS1 mutants lacking helicase and ATPase functions do not induce replication stress (64, 130). These host factors hijacked by parvoviruses may include those associated with MVM APAR bodies, including RPA and PCNA, which are required for MVM replication (69, 131, 132). The NS1 protein of MVM can transport the viral genome to the cellular DDR site through interaction with the viral genome and to heterologous DNA molecules containing NS1 binding elements, which may be involved in initiating viral replication (69, 119). MVM uses the formed APAR bodies as viral replication factories, in which NS1 interacts with the viral genome and DNA damage signaling proteins (such as DNA polymerase δ, MRE11, RPA) to achieve viral replication (39, 120, 131). B19V infection induces a DNA damage response with activation of all three PI3K kinases (126). B19V infection induced by the host cell DDR is critical to viral DNA replication through ATR and DNA-PKcs pathways (126). B19V recruitment of the PI3K kinase and its downstream effector molecules CHK2, CHK1, and KU70/KU80 to the viral DNA replication center and co-localize DNA damage sites (133) (**Figure 4**). Detailed studies, have found that NS1 protein regulates the transactivation of cellular genes through the TAD2 domain, which further mediates ATR activation and phosphorylates CDC25C at Ser216, which in turn inactivates the cell cycle protein B1/cycle protein kinase 1 (CDK1) complex and induced G2-phase arrest (94). MVC infection can only lead to the activation of ATM and ATR cascade, and only ATM activation is required to replicate the virus effectively and promote the proliferation of progeny viruses. AAV replication is recruited to the viral replication center of the helper virus (123) and induces a specific DDR regulated by DNA-PK (134) (**Figure 4**). DDR induced by NS1 protein induction depends on signal transduction by ATM kinase and leads to ATR-mediated signal inactivation, targeted degradation of p21 by proteasome, and inhibition of Cyclin B1 expression (117, 135). To ensure that DNA damage signals are transmitted and repaired effectively, the DDR protein must be in the right place at the right time. The NS1 of MVM can assist DDR-related proteins in locating DNA damage sites in cells (117) and induce diffusion to more DNA damage sites as replication progresses to achieve efficient viral replication (136) (**Figure 4**). AAV2 genomes and Rep78/68 also interact with sites of cellular DNA damage (123), and how NS1 or Rep78/68 is recruited to the DNA damage is unclear. Similar to AAV2 Rep78/68, the NS1-100/70 of HBoV1 induces a DDR (122). As with viral infection, NS1 induced DDR through phosphorylation RPA32 and H2AXduring the process (137), and NS1 can interact with Ku70 plays an important role in HBoV1 DNA replication in HEK293 cells (121). The NS1 protein phosphorylation of CPV induces DNA damage, cell cycle arrest at the G1/S phase, and cell apoptosis. NS1 is also associated with the mitochondrial outer membrane and DNA damage response, activating pro-apoptotic signals, Bcl2 and Bax, which generate oxidative stress (138).

### 4.2 Function and molecular mechanism of DDR in parvovirus infection and replication

NS1 is an essential nuclear localization protein, which enters the nucleus and plays its function in the nucleus under the action of nuclear localization signal or other intracellular proteases. It is an essential protein in the DNA replication process of parvovirus. DDR is a complex cellular regulatory network that continuously monitors genome integrity and can recruit various proteins or cytokines for signaling and repairing DNA damage. Studies have shown that multiple DNA virus infections can activate DDR (118), which viruses use to promote the replication of their genomes. Both NS1 and DDR responses play an essential role in parvovirus gene replication, and there may be some internal relationship or interaction mechanism between them to participate in parvovirus replication.

DNA damage caused by parvovirus NS1 can stimulate DDR by signal and rapidly recruit many host cell DNA replication and repair proteins to form virus-associated replicators (139). The NS1 of MVM can locate heterologous DNA molecules containing NS1-binding sequences to sites rich in proteins needed to repair and replicate damaged DNA and establish APAR bodies (131). In this process, NS1 may act as a bridging molecule to help the MVM genome to locate the cellular DNA damage site and improve the replication level and ability of the virus itself (23). L-Tag protein of polyomavirus can activate ATM and ATR pathways by regulating cellular transcriptional mechanisms, resulting in DDR response (140). HBoV infection of human respiratory epithelial cells cultured at the gas-liquid interface can induce significant DDR (141). DNA-PK activation is a critical DDR pathway for HBoV DNA replication (121).

## 5 Conclusions and future perspectives

Conclusively, the activation of the DDR pathway is crucial for the replication of parvovirus. However, using these routes might have unfavorable impacts that the virus has to overcome (110). Some studies have established a link between DDR signal and innate immune response (142). The depletion of PARP, BRCA2, RPA, and Rad51 can lead to the accumulation of cytoplasmic DNA, thereby activating the cGAS-STING pathway (142). Parvovirus inhibits interferon production in different ways; FPV NS2 and pBoV NP1 suppress the host IFN-β induction by targeting TBK1/STING and IRF9 (143, 144). Meanwhile, the replication of B19V and PPV is related to their virus proteins inhibiting interferon production (145, 146). The above results indicated that there is a complex interaction between DDR and the host’s natural immune response.

HBoV1 infection initiates a DNA damage response (DDR), activating all three phosphatidylinositol 3-kinase-related kinases (PI3KKs): ATM, ATR, and DNA-PKcs (122). Meanwhile, transfection of HEK293 cells with the double-stranded DNA genome or NS1 of HBoV1 also induced the phosphorylation of H2AX and RPA32, as well as the activation of all three PI3KKs (122). These results suggest that either HBoV1 DNA replication or NS1 expression can activate DDR. The MVM genome uses its essential non-structural phosphorylated protein NS1 to locate DNA damaged cell sites, thereby establishing viral replication centers (117). It is worth noting that the replication of AAV2 DNA, rather than the accumulation of single-stranded DNA genomes and the expression of Rep induced DDR in HEK293T cells (26), and It was confirmed that AAV2 DNA replication induces DDR, which in turn initiates the DNA repair process and partially promotes viral genome amplification (147). However, the specific causes of host cell DNA fragmentation caused by parvoviruses, whether it is DNA replication stress, NS1 nicking, cell cycle arrest, or induction of reactive oxygen species caused by the apoptotic pathway, are still controversial.

The parvovirus genome consists of the left end hairpin (LEH) and right end hairpin (REH), which play an important role in the virus replication process (148). In the process of viral replication, the hairpin transfer steps using the hydroxyl group (OH) of the terminal hairpin as a primer, including the synthesis and rearrangement of its palindromic ends, are essential for viral rolling-hairpin DNA replication (25). It has long been thought that the terminal hairpin sequence is essential for viral DNA replication. Qiu et al. found that the hairpin deletion duplex genome of HBoV1 replicates in HEK293, HBoV1 pathogenesis does not depend entirely on REH sequence to initiate rolling card DNA replication (148).These results suggest that hairpin structure is essential not necessary for parvovirus replication, but whether it is directly involved in parvovirus-induced DDR remains unclear. Our previous studies have found that NS1 can interact with host COPI-coated vesicles during PPV infection (149), thereby regulating the host cGAS-STING anti-infection immune pathway to promote virus self-replication. Subsequent investigations revealed that to facilitate the connection of NS1/COPϵ, NS1 first interacts with the host protein CCT5 (149). In the process of PPV infection, it was also found that NS1 can mediate the complete and incomplete autophagy reaction of host cells to meet the needs of different viral replication periods (150). However, the molecular mechanism of interaction among parvovirus replication, DDR, and anti-infection immune cGAS-STING is unclear.

As discussed in this review, the activation and regulation of ATM/ATR/DNA-PKcs DDR pathways play a crucial role in the life cycle of parvovirus. Parvovirus has evolved a mechanism using the DDR pathway to support virus replication, and the DDR induced by viral infection is of great significance in viral replication and pathogenesis (26, 151). In contrast to virus-induced DDR, host cell DDR can recognize specific hairpin-like structures in the viral genome and activate the host immune response to inhibit viral replication. In the long-term evolution, parvovirus has the function of selectively activating or utilizing host cell DDR, and it can selectively bind DDR components conducive to viral replication while eliminating harmful DDR components unfavorable to viral replication to complete its replication cycle efficiently. However, the selective mechanism of parvovirus infection and cellular DDR is still unclear, and the selective activation of different DDR molecules by different parvovirus infections remains a mystery. Parvovirus hijacking the DDR pathway allows the virus to obtain cellular resources for virus replication outside the S phase and promotes tolerance to replication stress caused by cell cycle changes. NS1 induces cellular DNA damage, but the biological significance of this damage for parvovirus replication using the DDR pathway is still unclear. In addition, the exact function of DDR protein in virus replication is still unclear. Understanding the mechanisms by which parvovirus and host DDR crosstalk will provide insight into the mechanisms of viral persistence and oncogenesis mechanisms and potentially identify therapeutic targets to treat parvovirus-associated diseases.

## Author contributions

SC: Writing – original draft, Writing – review & editing. FL: Writing – original draft. AY: Writing – original draft. KS: Writing – review & editing.
